# Production of marmoset eggs and embryos from xenotransplanted ovary tissues

**DOI:** 10.1038/s41598-023-45224-x

**Published:** 2023-10-24

**Authors:** Runa Hirayama, Hiroaki Taketsuru, Ena Nakatsukasa, Rie Natsume, Nae Saito, Shuko Adachi, Sayaka Kuwabara, Jun Miyamoto, Shiori Miura, Nobuyoshi Fujisawa, Yoshitaka Maeda, Keizo Takao, Manabu Abe, Toshikuni Sasaoka, Kenji Sakimura

**Affiliations:** 1 Department of Animal Model Development, Brain Research Institute, Niigata University, Niigata, 951-8585 Japan; 2 Department of Comparative and Experimental Medicine, Brain Research Institute, Niigata University, Niigata, 951-8585 Japan; 3 Department of Behavioral Physiology, Graduate School of Innovative Life Science, University of Toyama, Toyama, 930-0194 Japan; 4 Institute for Research Administration, Niigata University, Niigata, 950-2181 Japan; 5 Department of Behavioral Physiology, Faculty of Medicine, University of Toyama, Toyama, 930-0194 Japan; 6 Research Center for Idling Brain Science, University of Toyama, Toyama, 930-0194 Japan

**Keywords:** Biological techniques, Biotechnology, Developmental biology

## Abstract

The common marmoset (*Callithrix jacchus*) has attracted attention as a valuable primate model for the analysis of human diseases. Despite the potential for primate genetic modification, however, its widespread lab usage has been limited due to the requirement for a large number of eggs. To make up for traditional oocyte retrieval methods such as hormone administration and surgical techniques, we carried out an alternative approach by utilizing ovarian tissue from deceased marmosets that had been disposed of. This ovarian tissue contains oocytes and can be used as a valuable source of follicles and oocytes. In this approach, the ovarian tissue sections were transplanted under the renal capsules of immunodeficient mice first. Subsequent steps consist of development of follicles by hormone administrations, induction of oocyte maturation and fertilization, and culture of the embryo. This method was first established with rat ovaries, then applied to marmoset ovaries, ultimately resulting in the successful acquisition of the late-stage marmoset embryos. This approach has the potential to contribute to advancements in genetic modification research and disease modeling through the use of primate models, promoting biotechnology with non-human primates and the 3Rs principle in animal experimentation.

The common marmoset (*Callithrix jacchus*), a small primate, has attracted attention because of the need for primate models to analyze and develop treatments for human diseases, especially neurological diseases^1^. The marmoset is a small New World monkey that is not only relatively gentle and easy to handle, but also has a short gestation period and multiple (2–4) pups. Although the advent of primate genetic modification models is expected to help bridge the gap between research with rodents and actual clinical results^1^, there are many problems to actually perform genetic modification in marmosets even with the use of the latest gene-editing technology.

In the first place, to produce genetically modified marmosets, a large number of eggs are required. For oocyte retrieval, follicles are developed to mature by administering hormones for several consecutive days, and oocyte are harvested by a surgical technique, although a limited number of oocytes are obtained^1^. These procedures for continued collections of eggs lead to an animal ethics problem. As another issue, in order to obtain many eggs at a time, it is necessary to keep and treat multiple marmosets at the same facility, which makes the marmoset studies possible only at a limited number of large facilities with ample budget. Collection of the early-stage embryos was also reported to be available for gene editing through uterine perfusion after mating, but their number is limited^2^.

In the present study, we planned to use ovarian tissue taken from dead marmosets that died of natural diseases or accidents, or were euthanized for experiments, and were previously discarded without avail. Since those animals keep their ovaries remaining intact, we thought it could be useful and beneficial to make full use of their follicles and oocytes (Fig. 1a). For this project, we requested marmoset research facilities in Japan to donate ovaries from dead marmosets. However, since it is difficult to predict the timing and the number of ovaries to be donated, we first made a basic protocol for interspecies ovarian transplantation using rats, and then tried to apply this protocol to marmosets.

**Figure 1:**
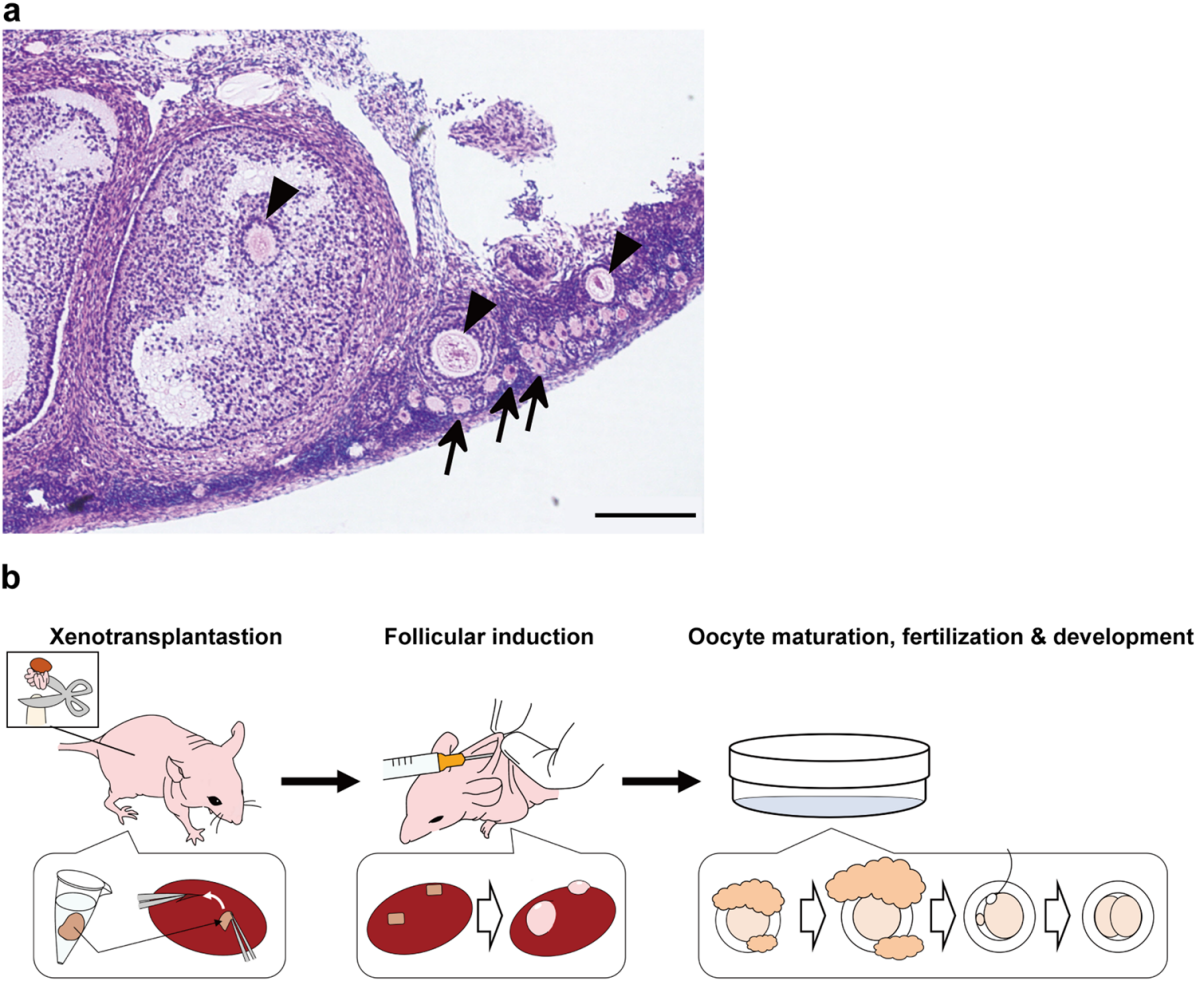
Image of marmoset ovary section and flow diagram of the study. (**a**) HE-stained adult marmoset ovary section for transplantation. Oocytes surrounded by cumulus cells within the follicle are indicated by the arrowheads (▲), and numerous undeveloped oocytes (↑) are seen on the cortical surface. Scale bar = 100 μm. (**b**) Flow diagram of the study.

Various approaches have been undertaken to develop methods for producing oocytes by xenotransplanting ovary tissues^3,4^. Many studies have focused on transplanting ovaries from different species into immunodeficient mice and using hormone treatment to induce the maturation and retrieval of oocytes from the transplanted tissue^5,6^. Successful in vitro fertilization and embryo retrieval have been achieved using collected oocytes from livestock such as pigs and cows^7,8^. Furthermore, in rodent studies, there is only one case reported on offspring production by transplanting mouse ovaries into immunodeficient rats and obtaining oocytes from them^9^. However, in xenotransplantation of primate ovaries, it has only been reported that follicles develop in response to hormones administered externally in the transplanted ovarian tissue^10,11^.

Here we report a novel method for transplanting marmoset ovaries into immunodeficient mice to obtain eggs, as well as a method for artificially fertilizing these eggs to make them develop into late-stage embryos (Fig. 1b).

## Results

### Preparation of recipient mice

Immunodeficient female mice of the KSN/slc nude strain were utilized as recipients for interspecies ovarian transplants. To mitigate the influence of endogenous hormones derived from the mouse ovaries, the ovaries were surgically removed when the mice reached 4 weeks of age, before attaining sexual maturity. At this stage, the vulvae were not opening, as shown in Suppl. Figure 1a. The renal capsule was chosen as a suitable site of ovary transplantation due to easy tissue fixation and a high rate of successful implantation as previously reported^12^. Ovary pieces, cut into 2–3 mm sections, were inserted under the renal capsule of the ovariectomized mice aged 8 weeks or older. The vulva that had been closed until transplantation started to open, once infiltration of blood vessels into the transplanted ovary was restored (Suppl. Figure 1b,c). Thus vaginal opening can be used as a good indicator of ovarian viability to facilitate follicle development in the transplanted ovary by adding hormone administration.

### Duration of rat ovary transplantation and protective effects of anti-apoptotic agents

**Figure 2:**
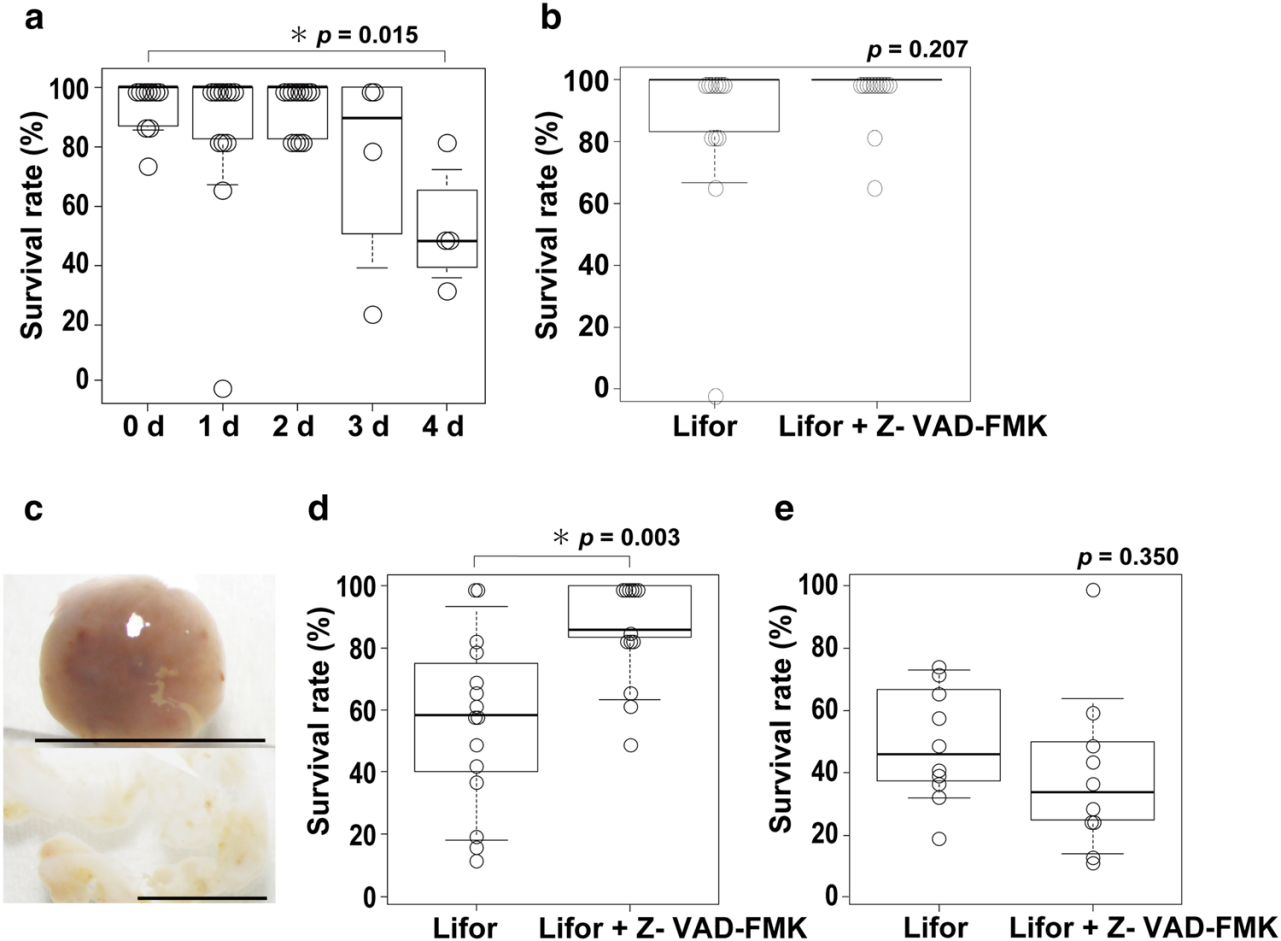
Effective preservation time and media for ovary transplantation. (**a**) Relationship between survival rate and the number of days after rat ovary collection. 0d: the number of mice = 10, the number of grafts = 72; 1d and 2d: the number of mice = 12, the number of grafts = 72; 3d and 4d: the number of mice = 4, the number of grafts = 18. (**b**) Relationship between survival rate of rat ovary and preservation media, Lifor and Lifor + Z-VAD-FMK. Rat ovaries were stored in each preservation medium for 1 day before transplantation. The number of mice = 12, the number of grafts = 72. (**c**) Marmoset ovaries; intact whole ovary (above) and cortical portion (below). Scale bar = 5 mm. (**d**) Relationship between survival rate of marmoset whole ovary and preservation media, Lifor and Lifor + Z-VAD-FMK. Lifor; the number of mice = 13, the number of grafts = 81; Lifor + Z-VAD-FMK: the number of mice = 15, the number of grafts = 113, p = 0.003. (**e**) Relationship between survival rate of marmoset cortical portion and preservation media, Lifor and Lifor + Z-VAD-FMK. Lifor; the number of mice = 10, the number of grafts = 85; Lifor + Z-VAD-FMK: the number of mice = 10, the number of grafts = 94, p = 0.350.

We investigated effective transplantation period of time for marmoset ovaries and protection effects of anti-apoptotic agents. Usually marmoset ovaries collected in other institutions were immersed in Lifor tissue preservation solution and refrigerated for delivery, during which the ovarian tissue may undergo deterioration. To determine effective period of time for transplantation, we conducted experiments using rat ovaries prior to marmoset ones. Ovaries were removed from 8-week-old or older SD female rats, divided into four portions, immersed in Lifor, and stored at 4 °C. Then the ovaries were cut into 2 mm squares and transplanted into recipient mice at different time points, from day 0 to 4 after removal, with day 0 being immediately after removal. The results indicated that there were no significant differences in deterioration between day 0 and up to day 2. However, on day 3, although not statistically significant, there was a decrease in implantation rate, and on day 4, the implantation rate was significantly lower (*p* = 0.015) (Fig. 2a), indicating that ovary transplantation must be perfomed within 48 h after removal.

Transplanted ovaries used in this experiment are stored and transported without blood flow after extraction. We considered that this ischemic condition during storage may induce apoptosis in oocytes and ovarian tissues, and investigated whether Z-VAD-FMK, which was previously used to inhibit apoptosis in frozen ovarian tissue, could prevent progressive deterioration of the ovaries over time. Rat ovaries, as prepared above were stored at 4 °C in either Lifor alone or Lifor supplemented with 50 μM Z-VAD-FMK. The ovaries stored with the addition of the anti-apoptotic agent showed a tendency to improve viability 24 h after storage compared to those stored with Lifor alone (Fig. 2b), although there were no significant statistical differences. We used 15 rats, 12 ovaries, 252 grafts in the first experiment, and 6 rats, 12 ovaries, 72 grafts in the second experiment, respectively, but the number of rats does not match with the total number (n = 15) due to the overlap.

### Effects of Lifor and anti-apoptotic agents on marmoset ovary protection

Marmoset ovaries transported from other research facilities are either a whole intact ovary or an ovary cortex alone from which oocytes were removed (Fig. 2c). Based on the results in the rat ovaries, marmoset ovaries were halved, put in both a tube with Lifor and the other tube with Lifor supplemented with 50 μM Z-VAD-FMK, and kept at 2–4 °C at each provider research institute before shipment. On arrival of the ovaries, they were treated with the transplantation procedure used for rat ovaries, and the results obtained from the two different tubes were compared. In the whole ovaries there was a significant difference in the implantation rate between the ovary preserved in Lifor and that in Lifor plus Z-VAD-FMK, whereas in the ovary cortex there was no significant difference between them (Fig. 2d,e). For ovarian protection experiments, we used 10 marmosets and 373 grafts.

### Transplantation conditions for marmoset ovaries

**Figure 3:**
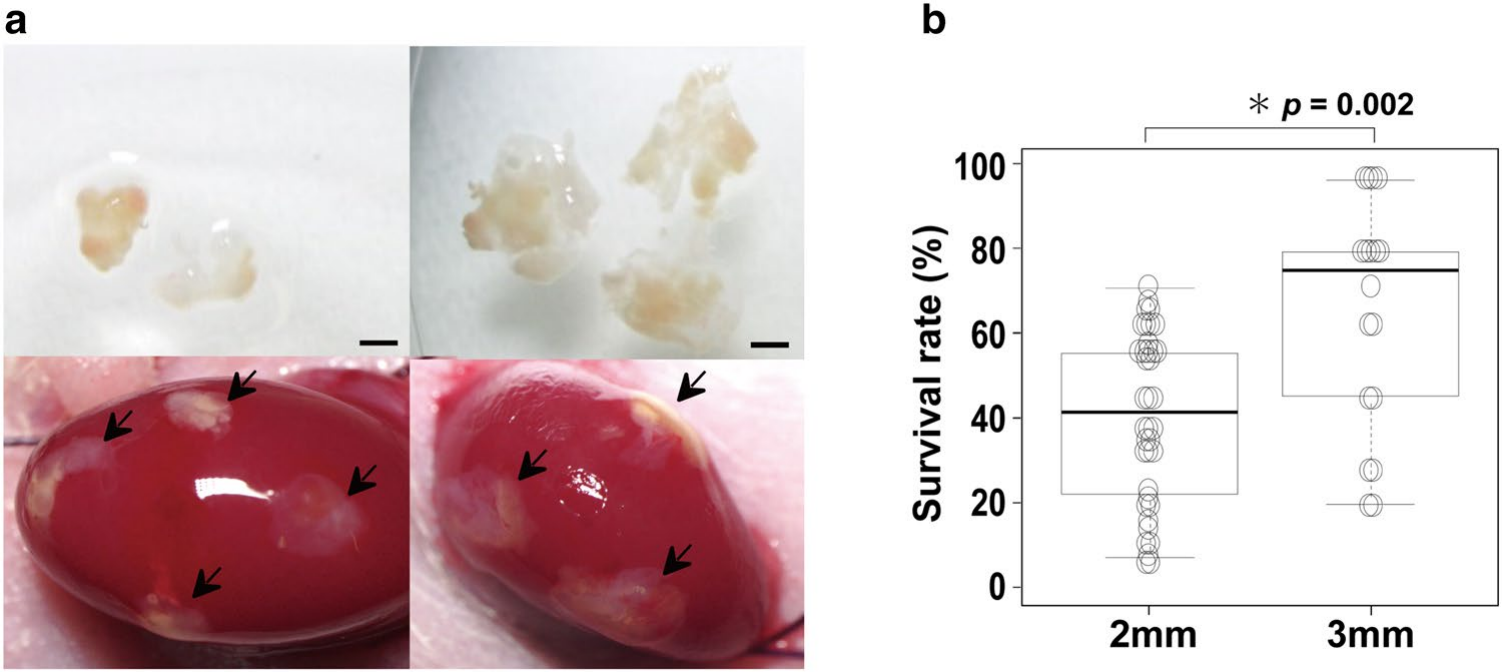
Graft size and survival rate in pieces of marmoset ovaries. (**a**) Marmoset ovaries with the sizes of 2 mm (upper left) and 3 mm (upper right) were inserted under the renal capsule and developed (lower left and right). Scale bar = 1 mm. (**b**) Their survival rates are shown. 2 mm: the number of mice = 38, the number of grafts = 328; 3 mm: the number of mice = 18, the number of grafts = 100, *p* = 0.002.

Like in the rat ovaries, marmoset ovaries were cut into 2 mm squares and transplanted to mouse kidney capsules. However, since their viability was lower than that of the rat ovaries (Fig. 2b,d,e), we tried to make the area touching to the kidney a little larger and raise the success rate by cutting the ovaries to 3 mm squares (Fig. 3a). The results showed the viability was significantly improved compared with that of the 2 mm squares (18 marmosets, 428 grafts, *p* = 0.002) (Fig. 3b). Further trial to make the size larger than 3 mm seemed technically unsuccessful, considering a small hole in about the 1 cm mouse kidney capsule.

### Conditions of hormone administration to develop follicles in the transplanted ovary

**Figure 4:**
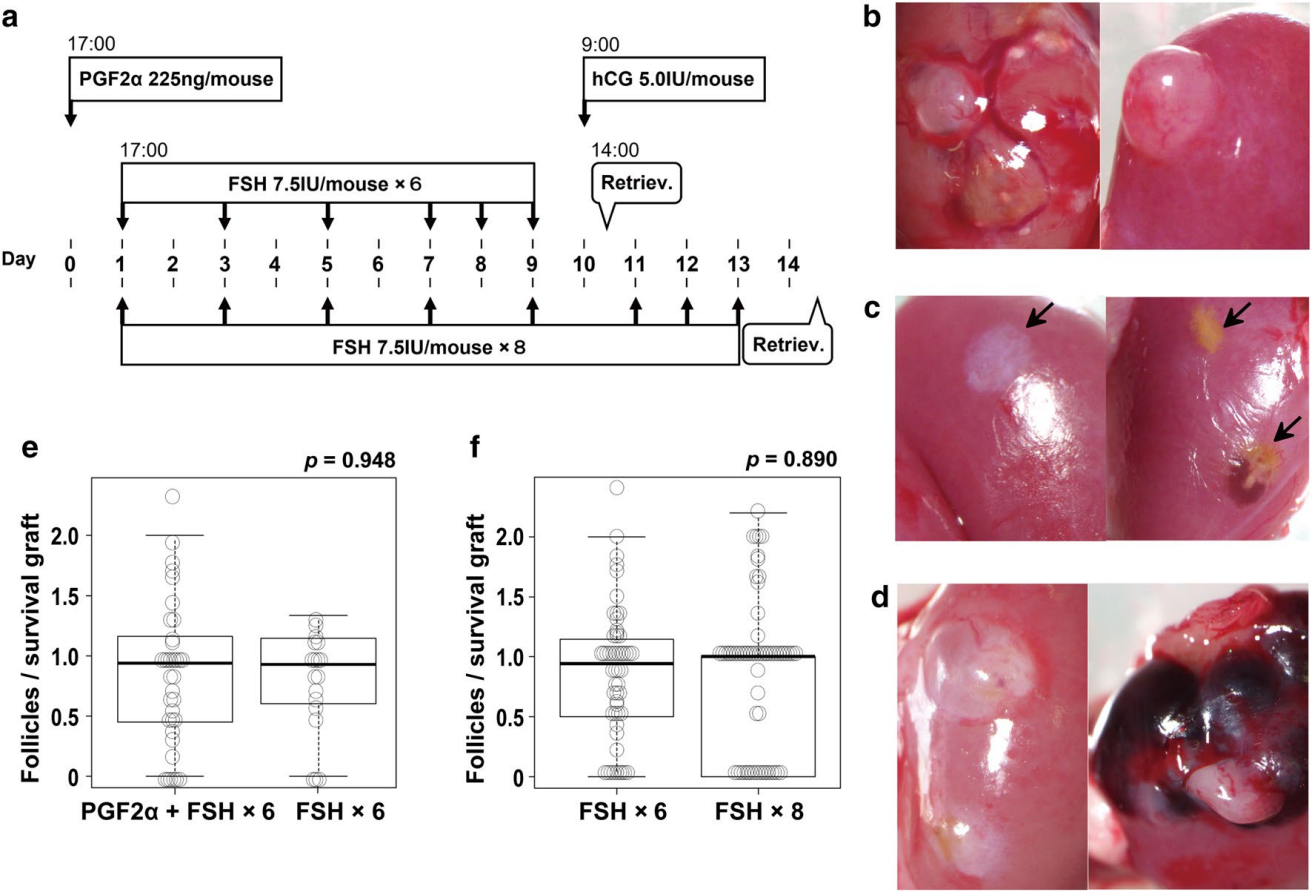
Follicle development in transplanted ovaries induced by various hormone administrations to mice. (**a**) Schematic diagram of various hormone administration tests. Thick arrows indicate the timing and frequency of intraperitoneal hormone administrations. Retriev.: graft and oocyte retrieval. (**b**) Neogenesis of blood vessels observed around the engrafted ovarian tissue (left) and developed ovarian follicle (right). (**c**) Unsuccessfully engrafted ovarian tissue showing fibrosis (left) and absorption scars (right). (**d**) Follicles from the untreated group (left) and those from the hCG-treated group showing intracavitary hemorrhage (right). (**e**) Effects of PGF2α + FSH (× 6) and FSH (× 6) administrations on follicle development. PGF2α + FSH × 6: the number of mice = 35 and FSH × 6: the number of mice = 18. (**f**) Effects of FSH administrations (× 6, × 8) on graft survival rate. FSH × 6: the number of mice = 53 and FSH × 8: the number of mice = 61.

Mice with transplanted rat ovaries needed 6.92 ± 3.62 days to open vulvae after transplantation, while it took mice with marmoset ovaries 21.17 ± 18.11 days. Since vulva opening was considered to indicate recovery of ovary function, hormones were administered after the sign of vulva opening to promote follicle development within the transplanted ovaries. Ovary tissues after hormone administration exhibited vascularization and bulging of developing follicles, as depicted in Fig. 4b. In contrast, unsuccessfully engrafted tissues showed fibrosis and absorption scars, as illustrated in Fig. 4c.

We started administrations from two doses of FSH and one hCG, based on the treatment for excessive ovulation in mice, but follicle development was unsatisfactory, thus changed to multiple doses of administration for longer period, similar to the method to increase oocyte retrieval in marmoset organisms. It has been reported that for marmoset ovarian retrieval, PGF2α is administered first to induce a follicular phase, followed by multiple doses of FSH administration for follicle growth, and finally hCG administration to obtain nearly mature oocytes^13^. Thus, in addition to 6 doses of FSH administration over 9 days, the same period for marmosets, we also tested 8 doses over 13 days, considering the fact that the body temperature of mice (37 ℃) is lower than marmosets (38.4–39.1 ℃) (Fig. 4a). It was assumed that low body temperature of mice would delay the development of marmoset follicles. These experiments revealed that PGF2α caused no significant effects (14 marmosets, 518 grafts, Fig. 4e), while hCG caused bleeding in the follicles, making oocyte retrieval difficult (Fig. 4d). Furthermore, increasing the number of doses and period of FSH administration from 6 doses over 9 days to 8 doses over 13 days caused no improvement in the number of follicles obtained (26 marmosets, 640 grafts, *p* = 0.890) (Fig. 4f). Thus we adopted 6 doses of FSH over 9 days as a most effective way of hormone administration to obtain as many follicles as possible.

### Conditions of maturation culture for retrieved marmoset oocytes

**Figure 5:**
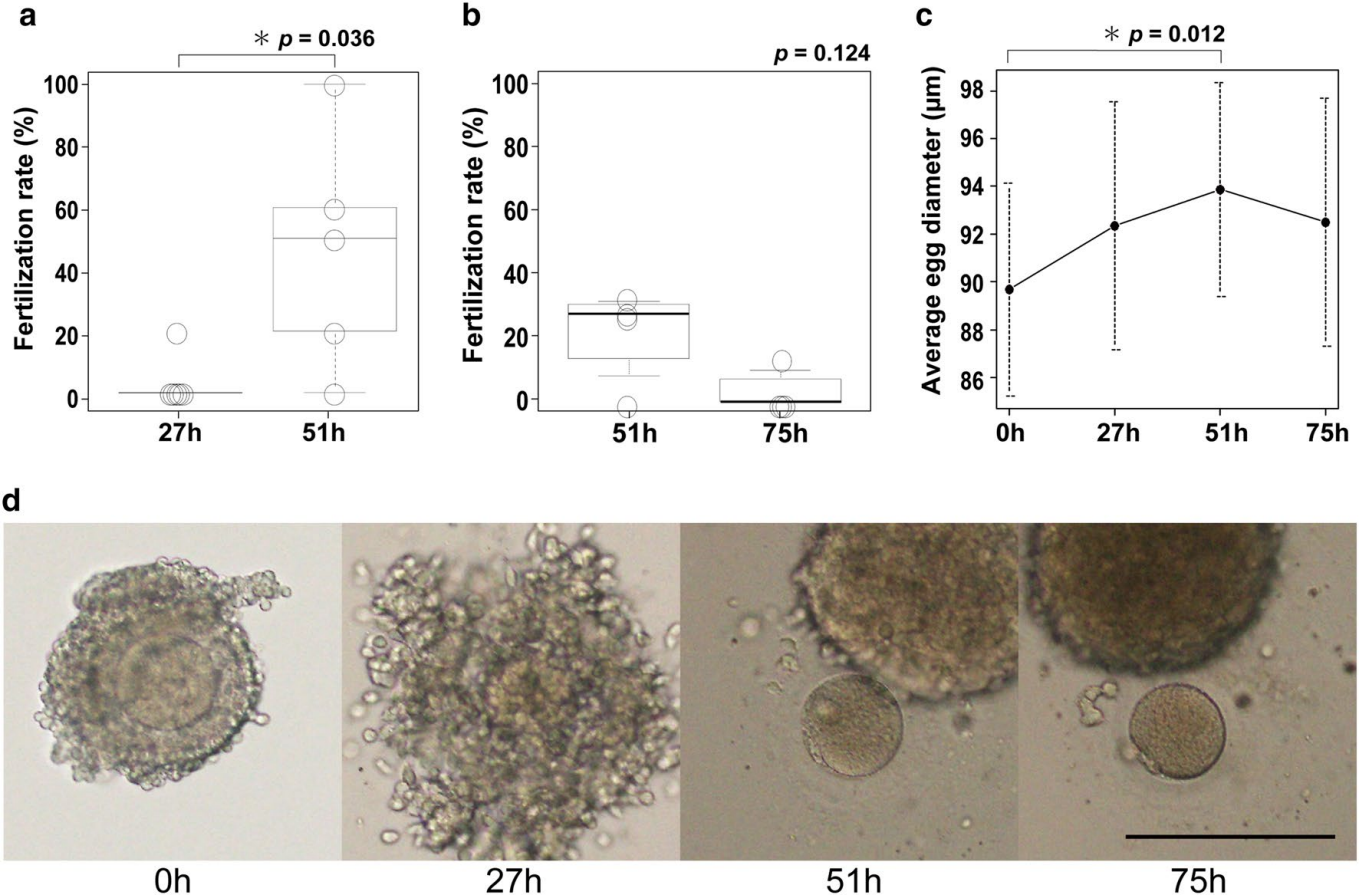
Effects of maturation culture time on fertilization rate. (**a**) Maturation culture performed at 37 ℃ with 5% CO_2_ for 27 h and 51 h. 27 h: the number of IVF groups = 5, the number of eggs = 25; 51 h: the number of IVF groups = 5, the number of eggs = 24,. p = 0.036. (**b**) Maturation culture performed for 51 h and 75 h. 51 h: the number of groups = 4, the number of eggs = 23; 75 h: the number of groups = 4, the number of eggs = 17. (**c**) Changes of egg diameters during maturation culture, the number of eggs = 15. (**d**) Morphological changes during maturation culture of retrieved oocytes. Oocytes are tightly surrounded by cumulus cells at 0 h that become swollen and start to detach from the oocyte 27 h later, and by 51 h almost separate from the oocyte. Scale bar = 200 μm.

In a previous study, oocytes retrieved from marmoset ovary tissues by excessive ovulation treatment were cultured for maturation for about 27 h^14^. We initially followed this procedure, but resultant fertilization rate of the eggs from transplanted ovaries was extremely low (about 10%). We next tested extension of the maturation time from 27 to 51 h, considering the difference of body temperature between mouse and marmoset. This extended period of 51 h resulted in significant improvement in the fertilization rate (4 marmosets, 225 grafts, *p* = 0.036) (Fig. 5a). Further extension of maturation time from 51 to 75 h was examined, but resulted in no further significant improvement in the fertilization rate, rather bringing about a tendency to lower fertilization rate (12 marmosets, 199 grafts, *p* = 0.124) (Fig. 5b). During temporal observation of the cultured oocytes, we noticed that they were growing with time (0 h vs 51 h; *p* = 0.012) (Fig. 5c), the largest in diameter at 51 h, 93.87 ± 4.47 μm, but did not reach the average size of mature marmoset, 100 μm, in diameter. We also observed the cumulus cells that were tightly surrounding the oocyte started to swell and got loose with time (Fig. 5d).

### In vitro fertilization and embryo development of marmoset eggs

**Figure 6:**
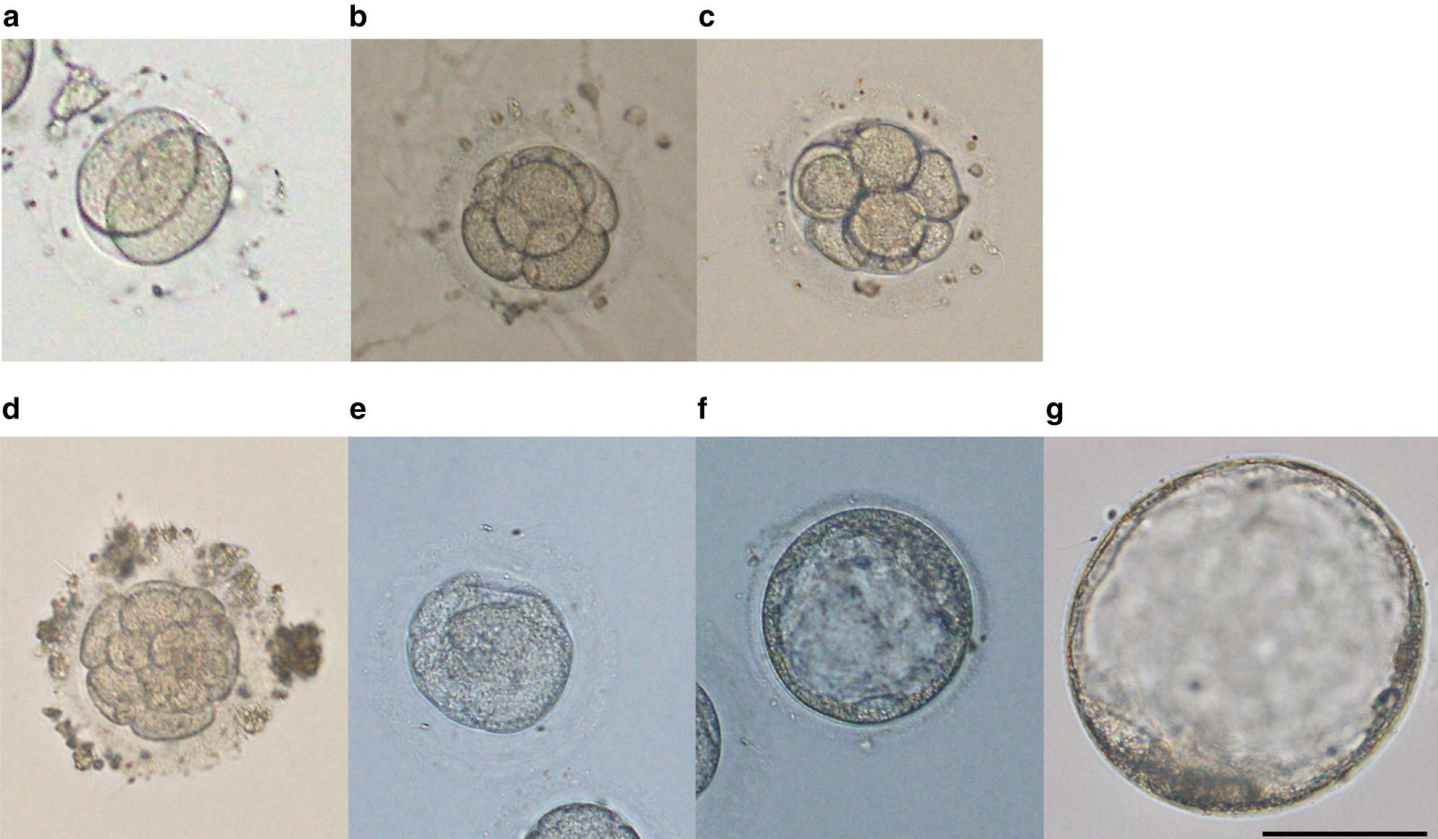
Progress of embryo development under atmospheric pressure, 37 ℃ conditions. (**a**) 2-cell (day 1), (**b**) 4-cell (day 5), (**c**) 8-cell embryos (day 6), (**d**) pre-compaction morula (day 5), (**e**) compacted embryo (day 6), (**f**) blastocyst (day 9) and (**g**) expanded blastocyst (day 11). Scale bar = 100 μm.

Matured eggs were fertilized by using ejaculated sperm from marmosets. We used 70 marmosets, 1449 grafts for grafts and oocytes collection experiments conducted with established protocols not as comparative experiments. Approximately 16 h after fertilization, the eggs were denuded and subjected to in vitro culture using human embryo culture medium, excluding degenerated eggs. In the earlier period of the experiments, many embryos ceased development at the two-cell stage. Fertilization was determined when the fertilized egg reached the 2-cell stage more than 16 h after the addition of sperm to the egg, because many eggs had swollen cumulus cells adhering around them, and polar body release could not be confirmed immediately after fertilization. However, by optimizing the ovarian transplantation conditions and hormone administration schedule, as well as minimizing the frequency of microscopic observations at atmospheric pressure and room temperature, the development progressed further than the two-cell stage. Actually, it became possible for 34.1% (31 embryos of 91 fertilized eggs) of the fertilized eggs to make progress to the late-stage embryos beyond the eight-cell stage, although some embryos exhibited developmental arrest, degeneration, or demise during culture. Furthermore, blastocyst development was successfully achieved by maintaining the culture for more than 10 days (Table 1 and Fig. 6).

**Table 1:** Results of fertilization: the number of eggs and percentage of the total oocytes from xenografted marmoset ovaries. Fertilized eggs were observed from day 2 after fertilization, day 4, and if necessary, days 8 and 10. The embryos showing cleavage were determined as fertilized eggs.

IVM eggs	Unfertilized eggs	Fragmented or dead eggs	91 fertilized eggs and these embryos developed to				
			2 cell	4 cell	8 cell	Morula	Blastocyst
444 (100)	225 (50.7)	128 (28.8)	44 (9.9)	16 (3.6)	16 (3.6)	10 (2.3)	5 (1.1)

### Physical conditions for embryo culture

**Figure 7:**
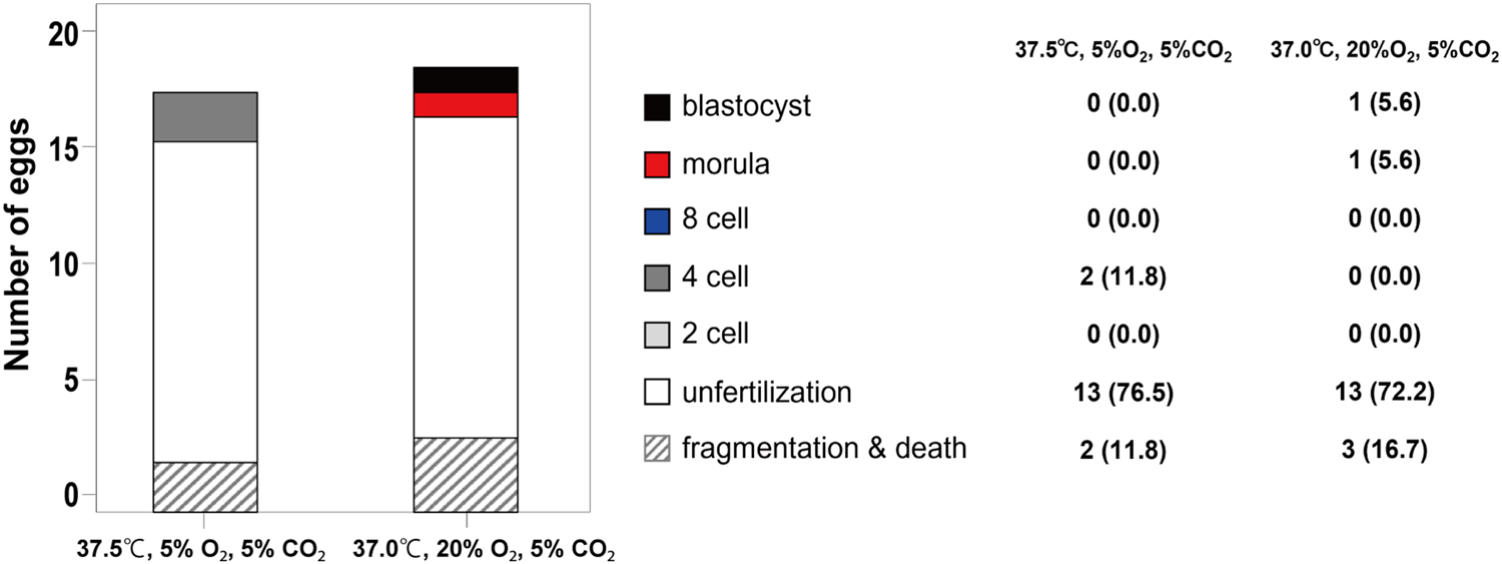
Comparison of culture conditions, low oxygen, high temperature conditions (5% O_2_, 37.5 ℃) and atmospheric pressure, 37 ℃. The atmospheric pressure, 37 ℃ condition allows a higher proportion of embryo development until the late stage.

Since low oxygen conditions are recommended in human and marmoset embryo culture^1,15,16^, we compared the condition for marmosets, 37.5 ℃, 5% O_2_, 5% CO_2_, with the standard culture condition of atmospheric pressure (20% O_2_), 37 ℃, and 5% CO_2_. We therefore examined the developmental efficiency of the embryos under the conditions in which the oxygen partial pressure was reduced to 5% and the temperature was slightly increased to 37.5 ℃. Under these conditions, developmental efficiency did not increase, rather decreased. (3 marmosets, 215 grafts, Fig. 7). One possible reason is the significant changes in oxygen pressure that occur during medium exchange or microscopic observation outside the incubator. As another reason, in our incubator it took a little longer to restore oxygen pressure because of opening and closing the door, which might have negatively affected embryo development. Given these conditions, the present experiment was conducted under atmospheric pressure, at 37 ℃ and 5% CO_2_, close to the mouse body temperature, and blastocyst development was successfully achieved.

## Discussion

In this study, we have succeeded in producing marmoset mature eggs that can be used for in vitro fertilization by xenotransplanting their ovaries to immunodeficient mice. The ovaries we used were provided by other marmoset research facilities in Japan. They were collected from marmosets who died of natural diseases, accidents, or euthanasia for experimental purposes, and had previously been disposed of. These fertilized eggs were able to make progress in vitro until the late-stage embryos suitable for uterine transplantation.

As recipients of transplanted marmoset ovaries, we used KSN/slc line mice, which were produced by introducing a BALB/c nude gene into DDD/1 mice. This line has a mutation that causes thymic defects and is immunodeficient with the reduction of T cells and thus capable of receiving foreign tissue. There have been reports using SCID mice as recipient mice, which are more severely immunodeficient than KSN/slc mice^5,17^. There is also a comparative study using BALB/c nude mice and C57BL/6 SCID mice, where goat embryonic ovaries were transplanted, concluding that angiogenesis in the transplanted ovaries was more evident in SCID mice, but no difference was observed in the number of follicles^18^. Since SCID mice are more difficult to maintain as well as expensive, we selected the nude mice to obtain oocytes. Although the eggs made progress to a blastocyst stage, its efficiency rate was not so high as we had expected. There might have been a contribution of the remaining immune system cells to the efficiency.

The conditions of the marmoset ovaries, either in the form of whole intact ovary or ovary cortex alone, are different from each other. In particular, at the provider research facility, the oocytes were surgically extracted from the ovaries and then only the ovarian cortexes were donated, indicating that some more time was required before the specimens could be transported to us. One of the purposes of this study is to transplant these ovary tissues efficiently into immunodeficient mice and make the remaining oocytes develop to a follicle stage. To determine effective conditions of ovary transportation, we studied the degree of deterioration with time in ovary tissues based on the results of rat ovary transplantation. Since the rat ovaries were shown to keep viability for two days when stored in Lifor at 4 ℃, we conducted transplantation of marmoset ovaries within 48 h after the removal. According to a previous report on implantation of human frozen ovary^19^, there were maintenance of primordial follicles and increased angiogenesis shown, when Z-VAD-FMK, pancaspase apoptosis inhibitor was contained in cryopreservation solution or administered to mice. We thus added Z-VAD-FMK to Lifor solution, and studied its preservation ability. While there was a significant improvement in a marmoset ovary tissue taken from a whole ovary, no significant difference was observed in ovary cortexes. This may be due to degeneration occurring in ovary cortexes after oocyte retrieval. Ultimately we used Lifor plus Z-VAD-FMK as a standard preservation solution.

As a suitable site of ovary transplantation into recipient mice, the subrenal capsule was selected. Although various sites were considered, including orbital and subcutaneous transplantation, renal subepithelial transplantation was superior in terms of efficiency and manipulation^12^, as previously reported. The kidney surface, where blood flow is plentiful, not only has a high tissue attachment rate, but also allows the tissue to be anchored by the capsule, which reduces the risk of losing small pieces of tissue. The size of the tissue grafts was also examined, and larger tissue grafts (2–3 mm square) had significantly higher tissue engraftment rates. We believe that vascular regeneration may have occurred more rapidly by increasing the area of adhesion of the tissue graft to the kidney^20,21^. We attempted to increase the size of the tissue strip to achieve stronger adhesion, but gave up because it was taking time to insert the tissue through a hole made in the small renal capsule. There are reports that vascular endothelial growth factor (VEGF) was administered to transplanted mice to improve the efficiency of angiogenesis^22,23^. Even in marmosets, the technique of immersing tissue fragments in VEGF solution before transplantation and administering VEGF to recipient mice before and after transplantation are worthy of consideration.

Mice normally have the vulva closed by the surrounding skin until sexual maturity, but it opens as ovarian function develops and estrogen is produced^5^. In the recipient mice, whose ovaries were removed in juvenile period, the vagina remains closed even after 8 weeks of age when they are fully grown. The mean vaginal aperture of mice transplanted with rat and marmoset ovaries was 6.92 ± 3.62 and 21.17 ± 18.11 days after transplantation, respectively. This difference in vaginal opening may be due to the difference in the sexual cycles between rats (4–5 days) and marmosets (about 28 days), rather than the one in the recovery of viable tissue. Vaginal opening after transplantation is caused by estrogen from the graft^5^, which is produced in the granulosa cells of the follicles^24^.

We tested many hormone administrations for follicle development in marmoset ovaries. Under our transplantation conditions, we were able to efficiently retrieve oocytes by administering FSH 6 times over a period of 9 days. Receptors for FSH and hCG are located in the cumulus cells and follicular membrane, while PGF2α, for which no effect was observed, has receptors in the corpus luteum. Since the corpus luteum is formed after ovulation of the mature oocyte, it is assumed that the marmoset ovaries transplanted into mice are unable to ovulate, and thus the corpus luteum is not formed either. For this reason, even if PGF2α is administered externally, no effect is seen. It is also assumed that although hCG administration causes ovulation-like phenomena in the follicles, its contents cannot be excreted because the ovaries are covered by the renal capsule, and blood clots are retained. Close examination of the blood clots occasionally found some degenerated oocytes, suggesting that the oocytes had already undergone an ovulation-like phenomenon and were deteriorating. How to make hCG effectively act may be essential to obtain more oocytes.

Oocytes collected from marmoset organisms are incubated for about 27 h for maturation before IVF. In our case, the fertilization rate of eggs collected from transplanted ovaries was greatly improved when incubation time for maturation was increased to 51 h. The size of the eggs, which was measured as an index of maturation, reached its maximum 90 μm in diameter at 51 h of incubation, but no further expansion was observed even when incubation time was increased to 75 h. These results indicate that oocytes derived from xenotransplantation are less mature than those obtained from living donors. Furthermore, the fertilization rate decreased when culture time was increased to 75 h in order to mature the oocytes, suggesting that deterioration may have occurred after passing the optimal time for fertilization. The size of eggs obtained from living organisms is approximately 100 μm in diameter, whereas eggs derived from xenotransplants were approximately 90 μm even after maturation culture. Other than the culture time, there must be other factors in culture conditions to make oocytes mature enough. From the data of this experiment, the optimal time interval for IVM is inferred to be 51 h, but it cannot be ruled out that the peak actually occurs before 51 h.

For human and primate oocyte and embryo culture, incubators with low oxygen concentrations (5%) are recommended to reduce oxidative stress. Marmosets have higher body temperatures (38.4–39.1 ℃) and their oocytes and embryos are often cultured at over 37.5 ℃^1,16^. We also cultured embryos under the hypoxic and high temperature conditions (5% CO_2_, 5% O_2_, 37.5 ℃), but the efficiency of embryonic development did not increase, rather decreased. The only method available at our facility was to perform microscopic examination under atmospheric pressure conditions, and we hypothesized that changes in oxygen partial pressure during transfer in and out of the hypoxic incubator affected the efficiency of embryo development. Furthermore, considering that transplanted ovaries have grown under the mouse body conditions, we used an incubator with atmospheric pressure conditions and mouse body temperature (5% CO_2_, 20% O_2_, 37 ℃) for culture. Even under these conditions, we were able to develop embryos to the late stage by minimizing manipulations outside the incubator, such as specimen inspection and medium exchange.

Our research method using otherwise discarded marmoset ovaries in the context of unrelated projects to develop oocytes in a mouse body, in vitro fertilize those eggs, and then finally develop them to late-stage embryos will promote biotechnology for non-human primates. This will also address the 3Rs (reduce, refine, replace) principle in animal experimentation, promoting animal welfare.

To our best knowledge, there have been no reports on generation of fertilizable primate eggs through heterologous transplantation. Thus this study could be groundbreaking, and leading to new possibilities.

## Methods

### Animals ethics and breeding conditions

All the animal experiments were conducted in compliance with the protocol which was reviewed by the Institutional Animal Care and Use Committee and approved by the President of Niigata University (Permit Number: SA01192). The study was carried out in compliance with the ARRIVE guidelines. Mice and rats were housed in plastic cages (polysulfone) in clean racks in a room temperature of 21–24 °C, humidity of 40–75%, ventilation frequency of about 15 cycles/h, and a 12-h light/dark cycle (light period 7–19 h), and were fed solid feed (Oriental Yeast Industry Co., Ltd.) and water ad libitum. Marmosets were kept in cages with W800 mm × D700 mm × H1601 mm (JIC Co., Ltd.), and with W870 mm × D660 mm × H1995 mm (CLEA Japan, Inc.) in a room with the temperature of 26–28 ℃, 40–60% humidity and a 14/10-h light/dark cycle (light period 6–20 h).

### Recipient mice

A total of 489 KSN/slc female nude mice, aged 4 weeks old, were purchased as immunodeficient mice from Japan SLC Co., Ltd., and bilateral ovary removal was performed within one week. At the age of over 8 weeks, rat ovaries were transplanted to 54 mice, and marmoset ovaries were transplanted to 435 mice.

### Removal of recipient mouse ovaries

The mice were anesthetized by intraperitoneal injection of a mixture of three anesthetics; 2.0 ml midazolam (5 mg/ml) (Midazolam, Sand Co., Ltd., Tokyo, Japan), 1.875 ml medetomidine hydrochloride (1 mg/ml) (Domitor, Nippon Zenyaku Kogyo Co., Ltd., Koriyama, Japan), and 2.5 ml butorphanol tartrate (5 mg/ml) (Vetorphale, Meiji Seika Pharma Co., Ltd., Tokyo, Japan) were mixed, and diluted fourfold with saline, before administration at a rate of 10 μl/g body weight. They were laid on their sides and incisions were made in the flank skin at the base of the hind legs, the subcutaneous fascia was cut open about 3 mm, the ovaries and upper uterus were withdrawn with tweezers, the upper uterus was burned off with a heat pen (Carving heat pen CH-1, Funtec, Kasukabe, Japan) for ovarian removal, and the skin was clamped with an autoclip (9 mm AUTOCLIP, Becton, Dickinson and Company, USA). After removal of both ovaries, the mice were injected with atipamezole hydrochloride (0.5 mg/ml; Antisedan, Nihon Zenyaku Kogyo, Koriyama, Japan) at a rate of 5 μl/g body weight for awakening on a 37 ℃ heating plate.

### Measurement of viable period of refrigerated ovaries

We examined how long ovaries removed from a living body remain ready for transplantation using rat ovaries. As donor rats, slc: SD female rats, homebred and Crl: CD rats purchased from Jackson Laboratory Japan, aged over 8 weeks were used (n = 15). They were anesthetized with CO_2_ inhalation and euthanized by cervical dislocation, then ovaries were removed. The removed left and right ovaries were each divided into four equal portions and stored in tissue preservation solution Lifor (Lifeblood Medical Inc., NJ, USA). During storage, they were kept at 4 °C in a tissue transport kit (CARD refrigerated transport kit, Kyudo Company, Japan), assuming marmoset ovary transport. The ovaries were implanted under the renal capsule of nude mice at 0, 1, 2, 3, and 4 days after the storage.

For ovarian protection experiments, Z-VAD-FMK (Caspase Inhibitor Z-VAD-FMK; G7231, Promega, USA) was added to Lifor (final concentration: 50 µM).

### Marmoset ovaries

Marmoset (n = 135) ovaries stored at 4 °C in the tissue transport kit, were delivered from nationwide research facilities in Japan. Detailed identity and quantity of many of the donated ovaries including the time lapse before they were sampled were unknown. Only those from animals that were sampled prior to rigor mortis were used. Their age range was 1–15 years. As in the case of rat ovaries, they were stored under the condition of immersion in Lifor or Lifor plus Z-VAD-FMK (50 μM).

### Ovary transplantation

Both rat and marmoset ovaries were transplanted with the same method. Ovariectomized immunodeficient mice anesthetized with a triad of anesthetics were laid on their side, and the subcutaneous fascia around the kidney was cut approximately 1 cm to expose the kidney. The kidney surface was wiped dry with a Kim wipe, and the renal capsule was incised about 1–2 mm using tweezers, then a glass capillary with a rounded tip was inserted under the renal capsule through the incised hole to create a pocket by peeling off the membrane (Suppl. Figure 2). Then 3–6 ovarian pieces, which were cut into 2–3 mm squares with a thickness of 1–2 mm in the Lifor beforehand, were inserted into the pocket under the renal capsule through the incision. Finally, the kidney was returned to the body, the fascia was sutured, and the skin was closed with autoclip. The animals were given Antisedan and placed on a heated plate until awakening.

### Induction of follicle development in the transplanted ovary by hormone administration

The amount of hormone to be administered to the mice to induce follicle development in the transplanted ovaries was determined through several preliminary experiments, referring to the literature on the administration of FSH to human ovarian transplanted mice^25^**.** To stimulate follicular development, the following hormones were administered to the mice transplanted with marmoset ovaries: 7.5 IU/mouse Folyrmon, follicle stimulating hormone (FSH) (Fuji Pharma Co., Ltd., Toyama, Japan), 225 ng/mouse Dalmadin, synthetic prostaglandin F2α injection (PGF2α; Kyoritsu Seiyaku Co., Tokyo, Japan), and 5.0 IU/mouse hCG, human chorionic gonadotropin (Fuji Pharma Co., Ltd., Toyama, Japan). FSH and PGF2α were administered at 17:00, hCG was at 9:00 a.m. next morning, and ovary fragments and oocytes were collected at 14:00. (Fig. 4a).

### Oocyte retrieval from transplanted ovaries

As preliminary arrangements for oocyte retrieval, a dish containing 2 ml of porcine oocyte-embryo collection medium (POE-CM; IFP1040P, Functional Peptides Research Institute, Yamagata, Japan) containing 10 IU/ml of sodium heparin (Mochida Pharmaceutical Co., Ltd., Tokyo, Japan), and another 2 ml POE-CM dishes for oocyte washing, were warmed on a heating plate at 37 ℃. Immunodeficient mice with transplanted ovaries were euthanized by cervical dislocation, and the ventral aspect was opened widely to quickly remove both kidneys. The transplanted ovarian fragments with visible blood vessels and swollen transparent follicles were confirmed as successful engraftment (Fig. 4b). The graft survival rate was calculated for each mice by (Number of survival grafts; exhibiting vascularization and bulging of developing follicles) / (Number of grafts visible on the kidney) × 100. We also calculated their survival rate by (Number of follicles) / (Number of survival grafts) × 100. When larger than 1,000 µm follicles were observed in the ovaries, they were separated from the kidney on an empty dish placed on a 37 ℃ micro-warm plate to avoid rupture, and only the follicles were collected. These follicles were transferred into a dish prepared beforehand for oocyte retrieval. Pressure was applied to the follicles in the solution with forceps, and a small hole was made with a 26-gauge needle to release the contents including oocytes, which were transferred with a capillary or a micropipette to a prepared dish for washing. After removing non-adherent cells, the oocytes were washed twice in maturation medium which had been pre-equilibrated under culture conditions, and served for maturation culture.

### Oocyte maturation culture

To induce maturation of the oocytes, maturation culture was performed. As culture medium, base of POM (IFT1010P, Functional Peptides Research Institute, Yamagata, Japan) was supplemented with 5% fetal bovine serum (ES FBS Thermo Fisher #10439024), 75 IU/ml FSH, and 10 IU/ml hCG, and filtered through a 0.45 μm filter. On this medium (100 μl) covered with HiGROW OIL Heavy mineral oil (Fuso Pharmaceutical Industries, Osaka, Japan), the oocytes were cultured in an incubator set at 37 ℃, 5% CO_2_, and 20% O_2_. The number of oocytes in an IVM drop was usually less than 10, with the maximum number not exceeding 20. To determine the optimal culture time, the oocytes were subjected to in vitro fertilization using ejected sperm, after 27-h-, 51-h- and 75-h-culture. Comparison of IVF data was made using the oocytes derived from the same marmoset ovaries.

### Sperm collection and in vitro fertilization of marmosets

For sperm collection, 4 male marmosets reared in our lab were used at ages over 1.5 years. The collection was conducted once a week on the day of IVF. When the collection was difficult, we switched to another animal avoiding prolonged stimulation and stress. As preliminary arrangements, two 1.5 ml tubes containing 500 μl of HTF medium (Transgenic Inc., Fukuoka, Japan) were equilibrated in an incubator at 37 ℃, 5% CO_2_, 20% O_2_. Sperms ejected by physical stimulation were put in the first HTF medium containing tube. After centrifugation at 3,000 rpm for 15 min at room temperature, most of the supernatant was removed except for a small amount, and the remaining sperm suspension was lightly pipetted. The suspension was transferred to a second tube, and then the second tube was centrifuged for 30 s at room temperature and placed in a 37 ℃, 5% CO_2_, 20% O_2_ incubator for 30 min. After incubation, active sperm that swam up to upper phase of the solution were collected and used for fertilization. The cultured eggs were transferred to 100 μl drops of HTF medium under oil and fertilized with the active sperms, 1–5 × 10^6^/ml for 16–17 h. These co-cultures occurred within a precisely regulated incubation chamber held at 37 ℃, with 5% CO_2_ and 20% O_2_.

### In vitro culture of embryos

Fertilized marmoset eggs were washed several times in Cleavage culture medium (83030010A, ORIGIO, Japan), and cultured in the same medium for 5 days. Embryos that made progress in egg division were selected and further cultured in Blastocyst culture medium (83050010A, ORIGIO, Japan) with 10% FBS. The fertilized rate was calculated for each group by (Number of the fertilized eggs) / (Number of used IVF eggs) × 100.

### Statistical analysis

Animals, oocytes and eggs were randomized into different groups with approximately comparable numbers of animals, oocytes and eggs in each group whenever possible. All data were tested for normality and homoscedasticity of the variances. The statistical analysis was conducted using the free statistical software R, version 1.61^26^. For the comparison between two groups, the Mann–Whitney U test was performed, and estimates were based on the median and quartiles. For comparisons involving three or more groups, the Kruskal–Wallis test was conducted, and multiple comparisons were performed using the Steel–Dwass method. For longitudinal data, a two-way repeated measures analysis of variance (ANOVA) was employed, and multiple comparisons were performed using the Bonferroni method. In all these data analyses, a significance level of p < 0.05 was considered statistically significant.

### Supplementary Information

Supplementary Figures.

## Data Availability

All data are available from the corresponding authors upon reasonable request.
