# Supplementary material for: Production of marmoset eggs and embryos from xenotransplanted ovary tissues
Source: Sci Rep. 2023 Oct 24;13:18196. doi: 10.1038/s41598-023-45224-x (PMC10598121; doi:10.1038/s41598-023-45224-x)
Supplement: Supplementary file 1 — Supplementary Figures. [file 41598_2023_45224_MOESM1_ESM.docx]

**Supplementary Information**

**Production of marmoset eggs and embryos from xenotransplanted ovary tissues**

Runa Hirayama^1,3,7^, Hiroaki Taketsuru^1,7^, Ena Nakatsukasa^1,8^, Rie Natsume^1^, Nae Saito^2^, Shuko Adachi^2^, Sayaka Kuwabara^2^, Jun Miyamoto^2^, Shiori Miura^2,4^, Nobuyoshi Fujisawa^2^, Yoshitaka Maeda^2^, Keizo Takao^3,5,6^, Manabu Abe^1^, Toshikuni Sasaoka^2*^ & Kenji Sakimura^1＊^

^1^Department of Animal Model Development, Brain Research Institute, Niigata University, Niigata 951-8585, Japan.

^2^Department of Comparative & Experimental Medicine, Brain Research Institute, Niigata University, Niigata 951-8585, Japan.

^3^Department of Behavioral Physiology, Graduate School of Innovative Life Science, University of Toyama, Toyama 930-0194, Japan.

^4^Institute for Research Administration, Niigata University, Niigata 950-2181, Japan

^5^Department of Behavioral Physiology, Faculty of Medicine, University of Toyama, Toyama 930-0194, Japan.

^6^Research Center for Idling Brain Science, University of Toyama, Toyama 930-0194, Japan.

^7^These authors contributed equally: Runa Hirayama and Hiroaki Taketsuru.

^8^Ena Nakatsukasa is deceased.

^＊^Email: [sakimura@bri.niigata-u.ac.jp](mailto:sakimura@bri.niigata-u.ac.jp), and [sasaoka@bri.niigata-u.ac.jp](mailto:sasaoka@bri.niigata-u.ac.jp)


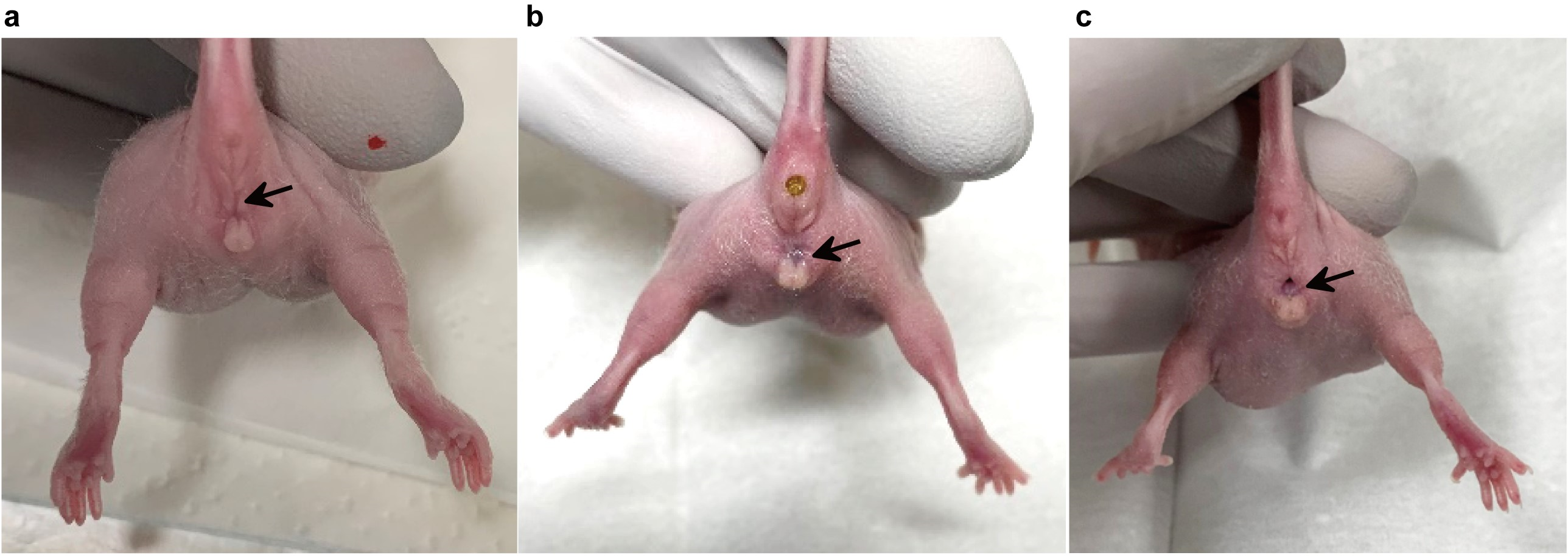


**Supplementary Figure 1.** Vaginal opening after ovarian live-implantation. The arrow indicates (**a**) a vagina of a 4-week-old mouse after ovarian removal, (**b**) a vagina of an 8-week-old mouse after ovarian transplantation, and (**c**) a vagina of a mouse over 4 weeks after transplantation, in which the transplanted ovary was viable.


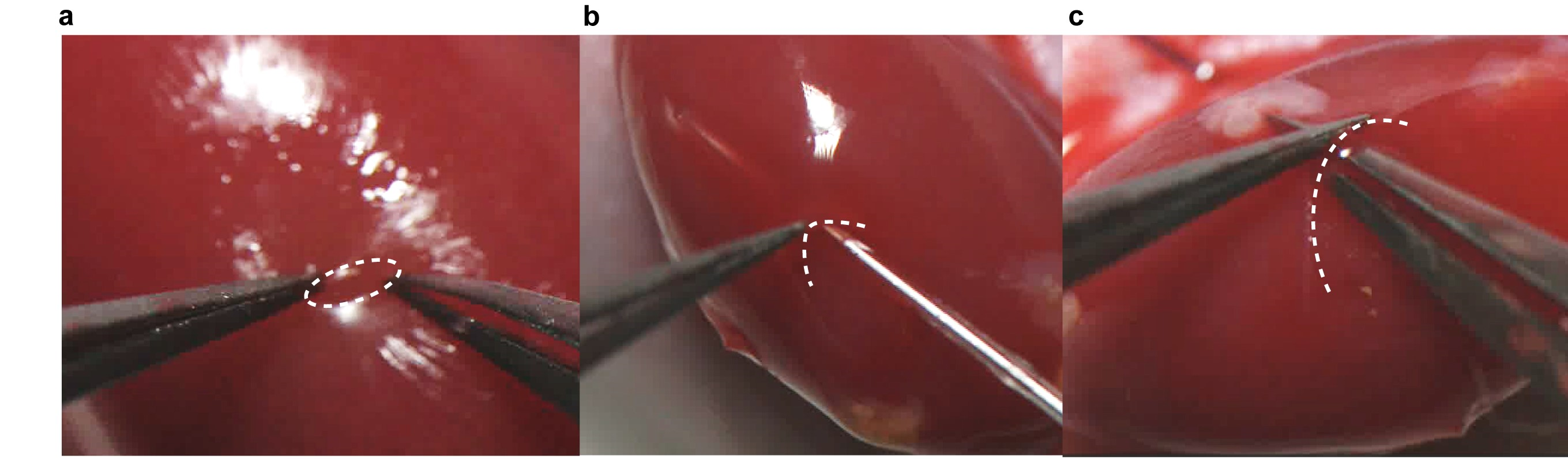


**Supplementary Figure 2.** Creation of an ovarian graft site on a mouse kidney. (**a**) A transplant pocket was made under the renal capsule by wiping the kidney surface dry, holding sharp tweezers with both hands, and making a 1-mm incision. (**b**) Through the hole, a glass capillary with a rounded tip was inserted to detach the renal capsule from the renal parenchyma taking care not to bleed. (**c**) The cut ovarian tissue is inserted into the pocket space through the hole.
